# On the Necessity of Validating Antibodies in the Immunohistochemistry Literature

**DOI:** 10.3389/fnana.2019.00046

**Published:** 2019-04-26

**Authors:** Laurent Gautron

**Affiliations:** Division of Hypothalamic Research and Department of Internal Medicine, The University of Texas Southwestern Medical Center, Dallas, TX, United States

**Keywords:** histology, peer-review, reagents, neuroscience, replicability

A lack of specificity is a very common problem with primary antibodies (including monoclonal antibodies), especially for those recognizing signaling proteins and receptors (Sim et al., 2004; Grimsey et al., 2008; Pradidarcheep et al., 2009; Egelhofer et al., 2011; Herkenham et al., 2011; Hafko et al., 2013; Solorzano et al., 2015; Bradbury et al., 2018). Sixteen years ago, Drs. Saper and Sawchenko published an article that explained the basics of immunohistochemistry (IHC) and provided guidelines aimed at helping scientists to determine the specificity of primary antibodies (Saper and Sawchenko, 2003). The two neuroanatomists called all primary antibodies with a specificity that was never properly verified and/or documented “magic antibodies” (Saper and Sawchenko, 2003). Then, academic journals published countless editorials, commentaries, and reviews on the problems with magic antibodies (Sim et al., 2004; Rhodes and Trimmer, 2006; Couchman, 2009; Kalyuzhny, 2009; Michel et al., 2009; Saper, 2009; Bordeaux et al., 2010; Hewitt et al., 2014; Baker, 2015; Bradbury and Plückthun, 2015; Uhlen et al., 2016; Weller, 2016). All of these articles conveyed a similar message: even though scientists use large panels of experimental controls to assess the specificity of their immunostaining, the controls are unfortunately seldom understood, performed or adequately documented. To make matters worse, basic information regarding the identification of antibodies is often missing in the scientific literature (Vasilevsky et al., 2013). These omissions do not mean that magic antibodies are all unreliable, but rather that their reliability cannot be evaluated. Moreover, without proper reagent identification, replicating IHC data is challenging. The good news is that the situation has noticeably improved in recent years and several academic journals, including the *Frontiers* journals, have adopted stricter editorial policies regarding antibody identification (e.g., Research Resource Identifiers; Saper and Sawchenko, 2003; McGrath, 2011; Gore, 2013; Hewitt et al., 2014). Moreover, several online and searchable databases now allow investigators to find detailed information regarding a large number of commercial antibodies (Helsby et al., 2014; Bandrowski et al., 2016). These changes show that reagent identification issues can be relatively easy to tackle through moderately constraining editorial policies. However, antibody identification is only the tip of the iceberg in regard to replicating IHC data: evidence of specificity should also be described. Based on an informal survey, the goal of this Opinion article is to start a conversation about editorial requirements for antibody validation in the *Frontiers* journals.

I conducted an informal survey on the publication rate of studies using antibodies with or without adequate identification and validation. I selected published articles in *Frontiers* journals with an emphasis on IHC, including *Frontiers in Neuroanatomy, Frontiers in Cellular Neuroscience, Frontiers in Neural circuits, Frontiers in Aging Neuroscience*, and *Frontiers in Neuroendocrine Science*. For convenience, I focused on the antibodies used for IHC in the field of neuroscience, even though magic antibodies affect all scientific fields employing antibodies and all antibody-based techniques (e.g., Western blotting). A total of 96 antibodies used for IHC data were categorized from randomly selected articles that were published between the years 2012 and 2018. Data from *Frontiers in Neuroanatomy* were compared to the combined data obtained from the other journals. Importantly, each antibody mentioned in the surveyed articles was categorized based on the criteria described below. The percentages of antibodies falling into each category are provided in Figure 1. Below is what I have learned from this small survey.

**Figure 1:**
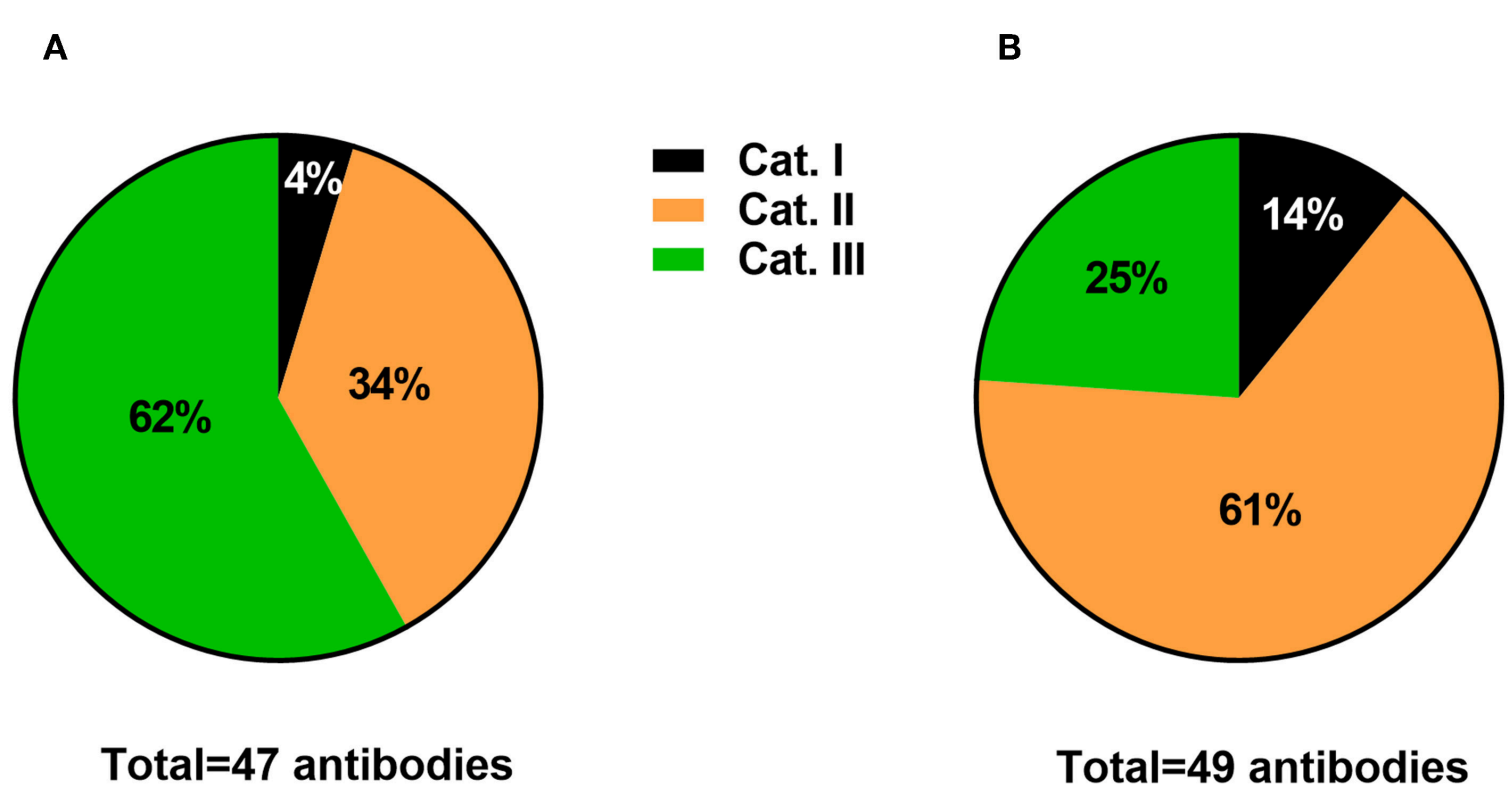
Informal survey of the publication rate of antibodies falling into 3 categories. Category I includes antibodies that were not satisfactorily identified. Category II includes antibodies that were adequately identified but lacked a convincing description of their specificity. Category III includes antibodies with proper identification and acceptable description of specificity. Data are expressed as the percentages of the total surveyed antibodies in *Frontiers in Neuroanatomy* **(A)** vs. other Frontiers in Neuroscience journals **(B)**.

Category I included antibodies with inadequate identification. Typically, these were antibodies that were difficult to identify based on the provided information (or lack thereof). For example, these were antibodies that were often listed without a catalog number, immunogen, host species, the concentration that was used, or no identification whatsoever other than the name of the epitope. As shown in Figure 1, Category I represented a small minority of antibodies used in studies from the surveyed journals (between 4 and 14%). This finding is hardly surprising considering that many articles have been written on the issue of magic antibodies that, with the exception of newcomers to research laboratories, most researchers submitting their work to *Frontiers* seem to be aware of the issues related to the use of magic antibodies.

Category II included antibodies with adequate information regarding manufacturing and usage but with little information regarding their specificity. Information regarding the vendor, host species, and the concentration that was used should have been included in the manuscript or easily findable on the manufacturer website. Antibodies in category II are listed without a description of their specificity in the examined tissue, location, and cell type. This category also included antibodies validated with inadequate control tests, such as omitting the primary antiserum. In the opinions of experts, the absence of immunostaining after omitting the primary is not a valid proof of specificity (Hewitt et al., 2014). Moreover, while most antibody manufacturers perform basic validation tests, they cannot possibly provide evidence of specificity for every application, cell type, and animal species. Experts insist that antibody validation is a tissue- and cell type-specific process, and each batch of antibody is different (Saper, 2005; Couchman, 2009; Holmseth et al., 2012; Hewitt et al., 2014). The publication rates of Category II antibodies were 34 and 61% in *Frontiers in Neuroanatomy* and the other surveyed journals, respectively (Figures 1A,B). The lower rate of Category II antibodies in *Frontiers of Neuroanatomy* can be explained simply by the fact that this journal publishes more fully validated antibodies, which I will explain further.

Finally, I included antibodies with complete identification and a description of the controls performed to establish specificity in Category III. The specificity of immunostaining may have been verified in the article itself or, at a minimum, in a prior study that is easily findable and cited. Based on previous recommendations (Saper, 2005; Couchman, 2009; Holmseth et al., 2012; Hewitt et al., 2014), what is considered a stringent control may include a Western blot of the tissue of interest, IHC of the tissue from a knockout animal, co-localization studies, and pre-adsorption studies, among other examples. Importantly, these tests are useful only if properly executed and do not guarantee absolute specificity. Thus, on the one hand, validating an antibody is admittedly a complicated, labor-intensive, and fallible process. On the other hand, it may not always be necessary to provide detailed validation controls in cases of antisera that label a molecule with a very well-known distribution pattern. The publication rate of Category III antibodies reached 62% in *Frontiers in Neuroanatomy* (Figure 1A). However, in journals other than *Frontiers in Neuroanatomy*, Category III antibodies only represented 25% of surveyed antibodies (Figure 1B).

In conclusion, our survey indicates that most articles published in Frontiers journals clearly identified the antibodies used for IHC. In contrast, descriptions of antibody specificity remain variable between journals. The present survey is admittedly small and has limitations. In particular, my categorization was not blinded and based on a limited set of criteria. In addition, I may have occasionally missed relevant information. However, beyond this survey, my personal experience as a reviewer for *Frontiers* is that antibody validation remains an issue. On several occasions, I had to request evidence of specificity from investigators who seemed caught off guard. Not only they were not aware of the necessity to validate antibodies, but they did not fully understand what constituted acceptable evidence of specificity. These requests likely resulted in unnecessary frustration and wasting time on both ends of the peer-review process. To help with this matter, I suggest a small change in the *Frontiers* guide for authors that consists of adding a description of the tests that were performed to validate antibodies used for IHC. The description could be similar to what other journals with a strong emphasis on IHC already request from their authors (Saper, 2005). Briefly, such a description should identify each antibody as precisely as possible and describe all the control experiments performed in the study or prior publications to ensure that the antibody detects its target. It should also include a detailed description of how the samples were prepared considering that a specific antibody may still give unsatisfactory results (i.e., false positive or negative results) on a piece of tissue has not been properly prepared. Claiming that all the necessary controls were performed with satisfactory results is not sufficient, and the relevant data and images should be included in the manuscript itself. Images of control experiments are extremely useful in helping evaluating the quality of an immunohistochemistry, especially the background levels generated by secondary antibodies. In addition, in the digital era, there is no shortage of space and it is easy to include control experiments, if necessary as supplementary data. Ultimately, it would be up to each individual reviewer and editor to decide what constitutes acceptable evidence of validation as well as what the length and content of the description should be. For instance, tissues from knockout animals are not always available. Moreover, it is not always necessary to characterize in depth an antibody that has been extensively used in the past. For the many investigators who already routinely include all the needed information and controls in their submissions, the suggested editorial requirement would make no difference. For the remaining investigators, such a small change in publishing requirements would likely accelerate the reviewing process, clarify editorial requirements, raise awareness on the problems with magic antibodies, and elevate the standards of the IHC literature in *Frontiers* articles.

## Author Contributions

The author confirms being the sole contributor of this work and has approved it for publication.

### Conflict of Interest Statement

The author declares that the research was conducted in the absence of any commercial or financial relationships that could be construed as a potential conflict of interest.
